# Low antiviral uptake of nirmatrelvir/ritonavir and molnupiravir in adult patients with COVID-19 in Taiwan in 2022

**DOI:** 10.7189/jogh.14.05032

**Published:** 2024-11-08

**Authors:** Fu-Der Wang, Phung-Anh Nguyen, David Lee, Bulent Taysi, Florence Lefebvre d'Hellencourt, Julia Spinardi, Phan Thanh Phuc, Whitney Burton, Yu-Hui Chang, Nguyen Thi Kim Hien, Shiue-Ming Lin, Yang Chieh, Moe H Kyaw, Jason C Hsu

**Affiliations:** 1 Division of Infectious Diseases, Department of Internal Medicine, Taipei Medical University Hospital, Taipei, Taiwan; 2 National Yang-Ming Chiao-Tung University, Taipei, Taiwan; 3 Graduate Institute of Data Science, College of Management, Taipei Medical University, Taipei, Taiwan; 4 Clinical Data Center, Office of Data Science, Taipei Medical University, Taipei, Taiwan; 5 Clinical Big Data Research Center, Taipei Medical University Hospital, Taipei Medical University, Taipei, Taiwan; 6 Research Center of Health Care Industry Data Science, College of Management, Taipei Medical University, Taipei, Taiwan; 7 Pfizer, Taipei, Taiwan; 8 Pfizer, Singapore; 9 Pfizer, New York, USA; 10 International PhD Program in Biotech and Healthcare Management, College of Management, Taipei Medical University, Taipei, Taiwan; 11 School of Pharmacy, College of Pharmacy, Taipei Medical University, Taipei, Taiwan; 12 Master Program in Global Health and Development, College of Public Health, Taipei Medical University, Taipei City, Taiwan; 13 College of Nutrition, Taipei Medical University, Taipei City, Taiwan

## Abstract

**Background:**

Antivirals are effective in reducing hospitalisation and death in mild-to-moderate coronavirus 2019 (COVID-19) patients. We estimated the antiviral uptake of nirmatrelvir/ritonavir and molnupiravir in adult patients with a syndrome coronavirus 2 (SARS-CoV-2) infection during the Emergency Use Authorization (EUA) period in Taiwan.

**Methods:**

A retrospective cohort study was conducted in Taiwan between January 2022 and December 2022. Patients aged ≥18 years with a SARS-CoV-2 infection were included from the Taipei Medical University Clinical Research Database (TMUCRD) and stratified in three risk groups according to World Health Organization criteria.

**Results:**

In total, 96 398 COVID-19 patients (mean age 46.7 ± 17.7 years, 45.8% male) were included. Of these patients 69.8% were classified as low risk, 29.8% as moderate risk, and 0.4% as high risk for progression to severe COVID-19. Nirmatrelvir/ritonavir was prescribed in 5.1% of the COVID-19 patients (low risk = 1.0%, moderate risk = 14.3%, high risk = 17.6%). Molnupiravir was prescribed in 1.9% of the COVID-19 patients (low risk = 0.1%, moderate risk = 5.8%, high risk = 6.9%).

**Conclusions:**

Nirmatrelvir/ritonavir and molnupiravir were poorly used in the treatment of adult COVID-19 patients in Taiwan during the pandemic in 2022, especially in moderate-to-high risk groups for progression to severe COVID-19.

Since its emergence in December 2019, coronavirus disease 2019 (COVID-19) caused by severe acute respiratory syndrome coronavirus 2 (SARS-CoV-2), led to significant morbidity and mortality worldwide. As of April 2024, Taiwan had nearly 10 million confirmed cases and 18 thousand deaths due to COVID-19 [1].

The Taiwan Food and Drug Administration issued an Emergency Use Authorization (EUA) on 17 January 2022 to allow the antivirals nirmatrelvir/ritonavir (Paxlovid®) [2] and molnupiravir (Lagevrio®) [3] for the treatment of mild-to-moderate COVID-19 in adults and children ≥12 years and weighing ≥40 kg who are at high risk for progression to severe COVID-19. Antivirals have potential to complement vaccination strategies by preventing the progression from mild-to-moderate COVID-19 to severe illness, particularly when administered within five days of symptom onset, and further reduce the burden on the health care system capacity.[4] The uptake of antivirals for COVID-19 in Asian countries is not known, but likely to be low due to limited availability and potential high costs.

We estimated the antiviral uptake of nirmatrelvir/ritonavir and molnupiravir in adult patients with a SARS-CoV-2 infection in Taiwan from January 2022 to December 2022, stratified by risk groups according to the World Health Organization (WHO) guidelines [4]. The rational for focusing on the antiviral uptake in Taiwan as the period of 2022 is of particular interest due to transition of a very well implemented national lock down to the opening of country boarders.

## METHODS

We conducted a retrospective cohort study in Taiwan based on the medical records of patients aged ≥18 years with a SARS-CoV-2 infection from the Taipei Medical University Clinical Research Database (TMUCRD) between January 2022 and December 2022. The TMUCRD includes electronic health record data from three affiliated hospitals: Taipei Medical University Hospital, Taipei Municipal Wanfang Hospital, and Taipei Medical University Shuang Ho Hospital [5]. Structured data (e.g. demographics, diagnoses, treatments, medication, discharge summary) and unstructured data (e.g. physicians notes, pathology reports) is pre-processed and validated into analysable research data and has been widely used for real-world evidence [5]. The study was approved exempt by Taipei Medical University Joint Institutional Review Board (TMU-JIRB number: N202310003) on 3 October 2023.

As of 2022, the TMUCRD has accumulated medical information of nearly 4.23 million patients across Taiwan. Data on adult patients with a positive SARS-CoV-2 PCR test in 2022 were retrieved from the TMUCRD. Patients aged <18 years or with missing gender and patients who received both nirmatrelvir/ritonavir and molnupiravir treatment were excluded. Corrections were applied to the data set for patients with multiple positive SARS-CoV-2 PCR tests and/or multiple prescriptions of antiviral treatment. Robust quality control measures were implemented and the data set was frozen before data analysis. The prescription period of antivirals is defined in the database as any time after a positive SARS-CoV-2 PCR test, but it is likely that most prescriptions were given within five days of symptom onset. Data was analysed in Microsoft Office Excel, with the latest available version by Microsoft, to calculate the uptake percentage of nirmatrelvir/ritonavir and molnupiravir in adult SARS-CoV-2 infected patients and stratified in risk groups. We used the updated WHO guideline on COVID-19 drugs to stratify COVID-19 patients into low risk, moderate risk, or high risk for progression to severe COVID-19 (**Table 1**) [4].

**Table 1:** Prescription rates of nirmatrelvir/ritonavir and molnupiravir in COVID-19 patients, Taiwan January 2022–December 2022

Variables	Number of COVID-19 patients	Antiviral prescription*	
	**Nirmatrelvir/ritonavir, n (%)**	**Molnupiravir, n (%)**
**All adult COVID-19 patients**	96 398	4884 (5.1)	1785 (1.9)
**COVID-19 patients stratified by WHO risk groups**†			
Low risk for progression to severe COVID-19†	67 305	705 (1.0)	99 (0.1)
Moderate risk for progression to severe COVID-19†	28 717	4113 (14.3)	1660 (5.8)
*Age ≥65 y*	16 823	3257 (19.4)	1320 (7.8)
*Obesity*	837	110 (13.1)	31 (3.7)
*Diabetes*	6397	1113 (17.4)	655 (10.2)
*Chronic obstructive pulmonary disease*	2026	265 (13.1)	139 (6.9)
*Chronic kidney disease*	2551	200 (7.8)	637 (25.0)
*Chronic liver disease*	2064	258 (12.5)	109 (5.3)
*Cancer*	3605	385 (10.7)	151 (4.2)
*Disabilities*	887	74 (8.3)	71 (8.0)
*Comorbidities of chronic disease*	15 469	2101 (13.6)	1167 (7.5)
High risk for progression to severe COVID-19†	376	66 (17.6)	26 (6.9)
**COVID-19 patients stratified by age**			
18–49 y	58 541	756 (12.9%)	147 (0.3%)
50–64 y	21 034	871 (4.1%)	318 (1.5%)
≥65 y	16 823	3257 (19.4%)	1320 (7.8%)
**COVID-19 patients stratified by hospitalisation**			
COVID-19 diagnosed in outpatients	95 106	4841 (5.1%)	1742 (1.8%)
COVID-19 diagnosed during hospitalisation	1292	43 (3.3%)	43 (3.3%)

COVID-19 – coronavirus disease 2019, WHO – World Health Organization

*Prescription period: any time after the positive syndrome coronavirus 2 (SARS-CoV-2 PCR test).

†The WHO guideline on COVID-19 drugs includes the following risk groups for progression to severe COVID-19: high risk (immunocompromised); moderate risk (≥65 years of age, obesity, diabetes, chronic cardiopulmonary disease, chronic kidney disease, chronic liver disease, active cancer, disabilities, comorbidities of chronic disease); and low risk (people who are not at high or moderate risk) [4].

## RESULTS

From January 2022 to December 2022, in total 115 699 COVID-19 patients were identified in the data set, 96 398 were adults aged ≥18 years and included in the study. Most patients (n = 19 206) were excluded for the reason age <18 years, four for missing gender, and 91 patients were excluded who received both nirmatrelvir/ritonavir and molnupiravir treatment. The mean age of the COVID-19 patients was 46.7 years (standard deviation (SD) = 17.7, 18–110 years) and 45.8% was male. According to the WHO guideline [4], respectively 69.8, 29.8, and 0.4% of the COVID-19 patients fell into the low, moderate and high-risk group for progression to severe COVID-19 (**Table 1**).

Overall, nirmatrelvir/ritonavir was prescribed in 5.1% of the COVID-19 patients and molnupiravir in 1.9%. Of the 67 305 COVID-19 patients in the low-risk group, 1.2% received nirmatrelvir/ritonavir or molnupiravir. In total 29 093 COVID-19 patients met the WHO criteria for moderate or high risk for progression to severe COVID-19, and thus were eligible for antiviral treatment. The uptake of antiviral treatment in this moderate-to-high risk group was low: nirmatrelvir/ritonavir 14.4% and molnupiravir 5.8%.

Immunocompromised conditions (i.e. high risk per WHO definition) were reported in 376 (0.4%) COVID-19 patients and nirmatrelvir/ritonavir or molnupiravir was prescribed in respectively 17.6 and 6.9% of these patients. In 16 823 (17.5%) elderly COVID-19 patients aged ≥65 years, nirmatrelvir/ritonavir was prescribed in 19.4% and molnupiravir in 7.8%. In line with the contraindication of nirmatrelvir/ritonavir in severe renal impairment [6], the prescription rate in patients with chronic kidney disease of molnupiravir (25.0%) was higher than nirmatrelvir/ritonavir (7.8%). Of the 1292 (1.3%) patients diagnosed with COVID-19 during hospitalization, 3.3% had antiviral nirmatrelvir/ritonavir prescriptions as well as 3.3% had molnupiravir prescriptions. The prescriptions of nirmatrelvir/ritonavir in 95 106 outpatients diagnosed with COVID-19 were higher (5.1%).

## DISCUSSION

Our data showed that nirmatrelvir/ritonavir and molnupiravir were poorly used in the treatment of adult COVID-19 patients in Taiwan during the pandemic in 2022, especially in moderate-to-high risk groups for progression to severe COVID-19. In 20% of the COVID-19 patients eligible for antiviral treatment based on the recent WHO recommendations [4], nirmatrelvir/ritonavir and molnupiravir were prescribed during the EUA period in Taiwan.

Low uptake rates of antivirals are reported also in other countries. Among adults in the USA a nirmatrelvir/ritonavir uptake of 14% and molnupiravir uptake of 1% was reported during December 2021–October 2022 [7], and respectively 28 and 4% for the period April 2022–June 2023 [6]. In Scotland, nirmatrelvir/ritonavir was prescribed in 19% and molnupiravir in 10% of people with COVID-19 at high risk of poor outcomes during December 2021–September 2022 [8]. Reasons reported for not prescribing antivirals in these patients include symptom duration longer than five days, lack of symptoms, low awareness of antivirals, concern about drug interactions, out-of-pocket costs, and lower priority for treatment due to the lower number of COVID-19 hospitalisations and deaths compared with earlier in the pandemic [6,7,9]. Nirmatrelvir/ritonavir has potential drug-drug interaction, mainly due to ritonavir CYP3A4 interactions. During the EUA period in Taiwan, the health care professionals have been given clear guidance from the Centres for Disease Control and Prevention on the criteria for antiviral prescription. We believe the barrier to antiviral prescriptions could be composed of a range of factors which includes time spent on assessing drug-drug interaction and potential benefit to risk awareness of health care professionals. The findings of our study warrant further investigation in subsequent research on the barriers of antiviral prescription in Taiwan.

In non-hospitalised patients with risk factors for severe illness, nirmatrelvir/ritonavir is the preferred oral treatment for mild-to-moderate COVID-19 and molnupiravir provides an alternative for adults for whom nirmatrelvir/ritonavir is contraindicated (e.g. due to drug-drug interactions or severe renal impairment) [6]. The treatment duration of both antivirals is five days and needs to be started within five days of symptom onset [10]. Early treatment with nirmatrelvir/ritonavir or molnupiravir is effective and cost-effective in reducing hospitalization and death in high-risk COVID-19 patients including the elderly, immunocompromised patients, and those with underlying medical conditions [10–15]. Nirmatrelvir-ritonavir demonstrated a greater risk reduction in hospitalization and death than molnupiravir compared to placebo [10]. In the EPIC-HR trial, administration of nirmatrelvir/ritonavir within three days after symptom onset resulted in a 89% reduction in 28-day hospitalisation and mortality rates in non-hospitalized and unvaccinated COVID-19 patients [13]. A similar result was shown in a Kaiser Permanente cohort study using real-world data; in non-hospitalised COVID-19 patients nirmatrelvir/ritonavir given within five days after symptom onset lowered the risk of hospitalisation and mortality with 80% up to 30 days [15]. Molnupiravir treatment within five days after symptom onset reduced the risk of hospitalisation or death through day 29 with 30% in at-risk unvaccinated adults with COVID-19 [14]. Two American studies showed the cost-effectiveness of nirmatrelvir/ritonavir and molnupiravir vs. best supportive care for outpatients with mild-to-moderate COVID-19 at risk for progression to severe disease [11,12]. In addition, nirmatrelvir/ritonavir and molnupiravir may have potential to reduce the risk of long COVID complications [16,17]. Since molnupiravir is less effective against COVID-19, it is recommended only for patients who are not eligible for nirmatrelvir/ritonavir [4,18].

The results are based on a single hospital network in Taiwan. As is generally known with studies based on registry data, caution is needed with data interpretation. For example, COVID-19 vaccine data in the TMUCRD is incomplete, since this is registered only when vaccinated at the three hospitals affiliated with the Taipei Medical University. The COVID-19 vaccine uptake in Taiwan was 79.8% on 31 December 2021 and 89.7% on 31 December 2022 [19]. Despite the nationwide COVID-19 vaccination programme the acute and long-term burden of COVID-19 remain substantial, especially in elderly and persons with underlying medical conditions [4]. Since COVID-19 patients who meet the moderate-to-high risk criteria of the WHO are at greater risk of hospitalisation and death from COVID-19, antivirals such as nirmatrelvir/ritonavir and molnupiravir are an important complementary tool to vaccination for the battle against COVID-19.

## CONCLUSIONS

Our study highlighted the missed opportunities to reduce the acute and long-term burden of COVID-19 in Taiwan using antiviral treatments. Early identification of COVID-19 patients with moderate-to-high risk criteria and treating with nirmatrelvir/ritonavir or molnupiravir within 5 days of symptom onset will have a substantial reduction in COVID-19 related short-term and long-term morbidity and mortality in Taiwan.
